# The Hospital

**Published:** 1918-03-30

**Authors:** 


THE
### INSTITUTIONAL WORKER
**Being a Special Supplement to "The Hospital"**

### OUR BUREAU OF INFORMATION.

### Rules for Correspondents.

1. Every letter must be accompanied by the coupon to be cut from the back cover (inside page) of The Hospital, current issue, and must contain the name and address of the correspondent with pseudonym for publication if desired. Replies by post cannot be given save under exceptional circumstances at the Editor's discretion.

2. Letters from Approved Homes in reply to special needs published in the Bureau should state terms and full particulars, and be sent prepaid under cover to the Editor of the Bureau with name written across coupon for identification.

3. Proprietors of Homes which have not yet been entered on the List of Approved Homes, but have spare accommodation likely to suit special needs, are invited to write for an application form for registration. The fee for registration, which includes two announcements of the Home in the Bureau and other privileges, is 10s.

4. All communications to be addressed to the Editor of The Hospital, 28 Southampton Street, Strand, London, W.C. 2, and marked "Bureau of Information."

### SUGAR BEET: ITS CULTIVATION AND USE.

### THE PREPARATION OF SYRUP.

Many hospital and institutional workers have exceptional opportunities of cultivating gardens and some are enthusiastic amateur gardeners. In these days the ornamental garden has been largely replaced by the kitchen garden, the value of which is very fully recognised by the institutional manager. How best to utilise the limited ground available is the question which enthusiasts will shortly be considering, and in view of the interest which has recently been taken in the cultivation and use of sugar beet we give some interesting facts in connection with the subject, for which we acknowledge our indebtedness to the *Amateur Gardener*.

#### What is Sugar Beet?

The roots from which sugar is extracted is a specially selected form of the wild beet of our sea-shores (*Beta vulgaris*) and is related to the red beet and the mangel. It has long, moderate-sized, parsnip-like roots, with a white flesh, grows deeper in the soil than the mangel, and has more fibres on its roots. Whereas in mangel roots the proportion of sugar varies from 3 to 8 per cent., in sugar beet the proportion of sugar averages 15 to 16 per cent. On the Continent—in Holland, Germany, Austria, Belgium, Russia, Switzerland, and Denmark—very special care is bestowed upon the selection of strains of sugar beet suitable for the varying soils and the climate.

#### How it is Grown.

As sugar beet is a deep-rooting plant it requires a deeply-ploughed or dug soil. Heavy clays, or soils with a clay subsoil, or peaty soils are unsuitable. All soils, too, must be free from stones. Stable manure should only be applied in the case of very poor soils, and then only in moderate quantities in autumn. Artificials, such as basic slag for the heavier soils, and superphosphate for the lighter ones; potash on light or medium soils is most essential, and nitrogenous fertilisers, like nitrate of soda or sulphate of ammonia, are generally used alone for sugar-beet culture. The seed has to be soaked for twenty-four hours in water, then sown in drills 27 in. apart. The seedlings should be subsequently thinned out to 9 in. apart. The best time to sow is about mid-April. The crop is ready to lift in mid-September. The lifting has to be done with very great care in order to avoid injuring the roots and consequently loss of sap containing the sugar.

The production of sugar from beet is a highly technical process, requiring not only special machinery, but technical knowledge and skill to carry it out successfully. All that the amateur can do is to extract a syrup possessing a certain degree of sweetness, but not equal in sweetening value to sugar. To enable our readers to do this we give the following instructive recipes published in the *Amateur Gardener*:—

#### Instructive Recipes.

(1) By Mr. A. J. Mortimer: First clean the sugar-beet roots thoroughly in clean water. Next cut the roots into thin slices or cubes and cover them with water, say, half a gallon to every 2 lb. of sliced roots. Boil slowly for one hour and simmer for one to two hours, or steam if steam is available, keeping the lid on the saucepan. When done strain off the liquor thoroughly and remove all scum or the syrup will taste of the beet. Evaporate the liquor in a gentle heat in an oven or copper until it is of the required consistency, then put into bottles for further use. The beet referred to in this recipe is Ryder's Saccharina.

(2) By Mr. A. Vernon Harcourt, of Ryde, Isle of Wight; First grow or for immediate use get your beet root. Any of the well-known seedsmen will supply seeds of the best sugar variety. Cut off most of the green top, and wash without breaking the outer skin. Boil for an hour, more or less, according to the size of the beet. Remove the rest of the top and the outer skin, which now comes off easily with the fingers. Weigh out 1 lb. Scrape each beet in turn with a vegetable grater. The heap thus formed must be handled lightly so that the little strips may remain separate. Heat 2½ pints of water to boiling-point in a saucepan about 6 in. across, and drop the beet into it. Heat again and boil gently for half an hour. Keep the cover on, guarding against frothing over. Filter the contents through a jelly-bag and squeeze them, collecting the syrup in a bowl. Repeat this operation, or take 2 lb. of beet and 4 pints of water. The 5 pints of weak syrup got thus will be acid and must be made slightly alkaline. A thimbleful of bicarbonate of potash dropped into the syrup effects this. Return the liquid to the saucepan and heat to boiling. At first there is frothing; when this is past boil quickly with the saucepan uncovered till half the water has boiled away. Take the temperature with a cook's thermometer, and let it rise to 217° F. Then pour, while still hot, into a wine bottle which has been heated with water or in the oven to a similar temperature. Cork well. The syrup is then sterilised and will keep. A dessertspoonful is equal to a lump of sugar.

The syrup serves well for puddings and stewed fruit, but, when used to sweeten tea or coffee gives a flavour, slight but discernible by our watchful sense of taste, and justly disliked. Of this the cause is doubtless some vegetable extract of which a small quantity accompanies the sugar, and it will be found that a solution of potassium permanganate, a famous oxidiser, added to the syrup little by little till the desired effect is produced, removes all flavour but that of sweetness.

Sugar beet may also be used as a cooked vegetable, and when served with white sauce is considered by many persons to be as good as, if not superior to, the ordinary red beet.

It must be borne in mind that sugar-beet syrup as produced by amateurs has not been proved by long experience to be an efficient substitute for sugar, and due consideration ought to be given to this fact in making a choice of food crops to be grown.

### MARCH QUESTIONS.

**(1) The Admission of Patients.**

**Is it an advantage to admit patients to small hospitals (under 50 beds) by subscribers' letters, or is the system of admission on a doctor's order only preferable?**

**(2) Repairs and Renewals.**

**Describe the system adopted at your institution for dealing with repairs, including (a) the arrangement in force for the periodical examination of the structure, and (b) the method of collecting, recording, and disposing of repair requisitions.**

### RULES.

The following rules must be observed:—

1. Contributions must be written on one side of the paper. Brevity and terseness are desirable features. The MSS. must bear the name and address of the sender and be accompanied by coupon to be cut from the back cover (inside page) of the current issue of The Hospital. A pseudonym must be chosen if the name is not to be published.

2. Contributions must be addressed to the Editor of The Hospital, Institutional Worker Supplement, 28 & 29 Southampton Street, Strand, London, W.C. 2, should reach him before the end of the current month, and be marked in the left-hand corner "Facts and Figures."

**A minimum payment of Five Shillings will be made for each published answer.**

### QUESTIONS INVITED.

In connection with our Question Box, we offer every month five shillings each for the two best questions which are sent in for consideration. The questions may be on any institutional subject and concern any department, from the secretary's to the porter's, or the matron's to the domestic staff; the one essential is that they must be practical and deal with points that have a definite relation to hospital or institutional administration, upkeep, finance, or management. Points relating to artificers' work, housekeeping, the laundry, out-patients, and other practical matters will be welcome. It is hoped that thus, when difficult or doubtful points arise in the course of their work, institutional workers will be encouraged to put them in the form of a question and send them to the Editor, The Hospital Bureau of Information, 28 & 29 Southampton Street, Strand, W.C. 2, marked "Question Box."

The best questions will be published in due course and our readers will be given the opportunity of answering them. Thus all institutional workers may be able to cooperate with the view of helping the work and smoothing the difficulties of each other. In short, we want the Question Box of the *Institutional Worker* to be freely used by every worker habitually.

### ENQUIRIES AND ANSWERS.

### *THE SICK AND IN NEED.*

**Case of Paralysis.**

If the case you mention requires treatment your best course would be to try for admission to one of the special hospitals for such cases—*i.e.*, the National Hospital for Paralysed and Epileptic, Queen Square, Bloomsbury, W.C., or the Hospital for Epilepsy and Paralysis, Maida Vale, W. On the other hand, if the case is a hopeless one, a home would be more suitable. Perhaps you will send us fuller particulars, when we would be better able to help you.—Anxious.

### *WAR MATTERS.*

**Literature for the Wounded.**

If you do not take advantage of the Post Office scheme of sending literature to the soldiers, the London Library will be glad to receive it for distribution to those of our Navy and Army suffering from wounds and disease. The valuable books are sold to buy larger numbers. The books are sorted at the London Library, and then given to the Red Cross War Library, Surrey House, Marble Arch. Parcels should be addressed to the Librarian, London Library, St. James's Square, London, S.W. 1, and clearly marked "For Wounded."—Kitty.

**Information Concerning Prisoners of War.**

Inquiries are made and information is given by the International Agency for Prisoners of the War of the International Red Cross Committee, Geneva, gratuitously. The only occasion upon which a charge is made is when an inquiry is accompanied by a request for an answer by telegram.—O. W.

**Radiographer's Assistant.**

We regret we do not know of a vacant post such as you desire. You might write to the Secretary of the British Red Cross Society, 83 Pall Mall, S.W., stating your wishes. Also study the advertisements which appear in the medical and nursing journals from time to time.—R. P. H.

### *EMPLOYMENT AND TRAINING.*

**Training as Mental Nurse.**

From twenty to thirty is the usual age to commence training in an asylum for the insane. You will find a complete list of training-schools for mental nurses in "How to Become a Nurse," published by The Scientific Press, Ltd., 28 and 29 Southampton Street, Strand, W.C. 2, price 2s. 10d. post free.—Bunny.

**Position in Munition Factory.**

We suggest that you enter your name at your nearest Labour Exchange; also apply to the Ministry of Munitions, Whitehall Court, S.W. 1.—Nurse C.

**Pharmacy for Ladies.**

You probably mean the Westminster College for Lady Dispensers. You must send your application for particulars to the Secretary, 112 St. George's Road, Southwark. Enclose a stamped envelope for reply.—Bee.

**Dentistry for Ladies.**

The prospects for women dentists in this country are certainly improving. A course of four years is necessary to obtain the diploma of the Royal College of Surgeons in dental surgery; the first two years may be carried out at the London School of Medicine for Women in connection with the Royal Free Hospital, Gray's Inn Road, London, but the dental studies must be taken at a dental hospital. Women students are received at the National Dental Hospital, Great Portland Street, London.—Edith M.

**"War" Probationers.**

Amongst the hospitals in London which receive "war" probationers are: The London Hospital, Guy's Hospital, Charing Cross Hospital, and St. Mary's Hospital, Paddington. Your application should be made to the Matron in each case.—Sesame.

**Travellers' Aid Society.**

If you will write to the Travellers' Aid Society, 3 Baker Street, London, W., they will no doubt send a representative to meet your daughter who is coming to London to undertake war work and who is without friends to meet her; they will also see her to her destination.—Mother.

**Services to the Wounded.**

We could give you no assurance that you would obtain work among the wounded abroad, and we suggest that you write to the British Red Cross Society, 83 Pall Mall, London, S.W., unless you prefer to join one of the Services, when you would, of course, have to go wherever you were sent.—T. M.

### *MISCELLANEOUS.*

**Fixation in Radiography.**

There are so many different head-fixing devices to facilitate radiography of the head in various positions that it is extremely difficult to suggest any special make, for different radiographers have varying views as to what is most useful in this respect. The Martin-Berry chair, manufactured by Watson and Sons, of 196 Great Portland Street, presents many advantages. Your best course is to get the makers to give you a practical demonstration.—Radiographer.

**The Editor will be glad to receive correspondence and to consider contributions upon all subjects relating to institutional work which affect the welfare of institutional workers.**

